# The complete plastid genome of an Antarctic moss *Chorisodontium aciphyllum* (Hook. f. & Wilson) Broth (Dicranaceae, Dicranales)

**DOI:** 10.1080/23802359.2021.1909435

**Published:** 2022-04-22

**Authors:** Zhaojie Ren, Hongfeng Chen, Suzhou Zhang

**Affiliations:** a Nature Department, Shandong Museum, Jinan, China; b South China Botanical Garden, Chinese Academy of Sciences, Guangzhou, China; c Fairylake Botanical Garden, Shenzhen & Chinese Academy of Sciences, Shenzhen, China

**Keywords:** *Chorisodontium aciphyllum*, mosses, plastome

## Abstract

Plastid genomes are useful markers in resolving plant phylogenetic relationships for various taxonomic groups. Here, we sequenced and *de novo* assembled the complete plastid genome sequence of an Antarctic moss *Chorisodontium aciphyllum* (Hook. f. & Wilson) Broth using genome skimming data. The newly generated plastid genome is conserved in structure and gene content compared with that of other Bryopsida. Plastid phylogenetic analysis of mosses recovered a robust phylogeny in which *Chorisodontium aciphyllum* clustered with *Fissidens nobilis* from the Dicranales. The plastid genome sequence of *C. aciphyllum* will aid future evolution and diversification studies of land plants.

Antarctica, characterized by low temperatures, high aridity and ultraviolet radiation, and strong thermal excursions, is considered as an extreme environment on earth (Pugh 1980). King George Island is the largest of the South Shetland Islands belonging to the maritime Antarctic zone (Lee et al. 2008), where the ameliorating effect of the ocean produces a milder climate (Kanda and Komarkova 1997). King George Island is dominated by a diversity of bryophyte (especially mosses) and lichen species with only two vascular plant species (*Deschampsia antarctica* Desv. and *Colobanthus quitensis* (Kunth) Bartl. (Øvstedal and Lewis-Smith 2002). The moss *Chorisodontium aciphyllum* (Hook. f. & Wilson) Broth (Dicranaceae, Dicranales) is widespread in the northern maritime Antarctic and its regrowth was observed from a cold-based glacier after about 400 years of ice cover (Roads et al. 2014). Here, we report the complete plastid genome of *C. aciphyllum* to document its genome structure, evolutionary history, and to contribute to the study of Antarctic bryophytes.

Fresh materials of *C. aciphyllum* were collected nearby the Chinese Antarctic Great Wall Station located in the south of King George Island (add coordinates here). The voucher specimen has been deposited at SZG (Herbarium of Shenzhen Fairy Lake Botanical Garden, Shenzhen, China), under collection number of Yao655 (Yang Liu, yang.liu0508@gmail.com). DNA extraction was performed with the NucleoSpin Plant II Midi DNA extraction kit (Macherey-Nagel, Düren, Germany). The DNA quality and quantity were examined using 1% agarose gel electrophoresis, Qubit fluorometer (Invitrogen, Carlsbad, CA), and NanoDrop 2000 spectrophotometer. For genomic DNA sequencing, 1 µg high quality genomic DNA was sheared using the Covaris M220 (Woburn, Boston, MA), DNA fragments 300–500 bp were selected to generate the sequencing libraries using the Illumina TruSeqTM DNA library preparation kit (Illumina, San Diego, CA) following the manufacturer’s instructions. The libraries were paired-end (2 × 150 bp) sequenced on an Illumina HiSeq 2000 sequencing platform at the Novogene (Beijing, China). Approximately, 3 Gb sequencing reads were generated for the sample, and the sequence reads were submitted to the GenBank SRA database under accession number SRR13239595. The raw NGS data were trimmed and filtered for adaptors, low quality reads, undersized inserts, and duplicate reads using Trimmomatic (Bolger et al. 2014). The resulting clean reads were *de novo* assembled using CLC Genomics Workbench v5.5 (CLC Bio, Aarhus, Denmark). The assembled contigs were Blast searched against the plastid genome sequences of *Fissidens nobilis* (GenBank accession no.: NC_044155), yielding a complete circular plastid genome sequence of 123,853 bp with an average read coverage of ∼175×. The plastome of *C. aciphyllum* was annotated using PGA software (Qu et al. 2019) with the reference plastome sequence of *Fissidens nobilis* and submitted to GenBank in Geneious v10.0.2 (www.geneious.com) under the accession number of MW355440.

The complete plastid genome of *C. aciphyllum* is 123,853 bp in length with an overall GC content of 29.8%. The genome displays a typical quadripartite structure consisting a small single-copy region (SSC; 18,673 bp), a large single-copy region (LSC; 86,076 bp), and a pair of identical inverted repeats (IRs; 9,577 bp). The genome encoded a non-redundant gene set similar to that of the other mosses in Dicranales, including 82 protein-coding genes, four rRNA genes, and 31 tRNA genes. Nine protein-coding genes (*atpF*, *ndhA, petB*, *petD*, *rpl2*, *rpl16*, *rpoC1*, *ycf3*, and *ycf66*) were disrupted by one intron, and two genes (*clpP*, *ycf3*) were broken by two introns.

Phylogenetic analysis of the plastid genome of *C. aciphyllum* is was performed using 27 plastomes of mosses currently available from the NCBI Organellar database (https://www.ncbi.nlm.nih.gov/genome/browse/?report=5#!/organelles/mosses). Each of the conserved 82 protein-coding genes (*accD*, *atpA*, *atpB*, *atpE*, *atpF*, *atpH*, *atpI*, *cemA*, *chlB*, *chlL*, *chlN*, *clpP*, *infA*, *matK*, *ndhA*, *ndhB*, *ndhC*, *ndhD*, *ndhE*, *ndhF*, *ndhG*, *ndhH*, *ndhI*, *ndhJ*, *ndhK*, *petA*, *petB*, *petD*, *petG*, *petL*, *psaA*, *psaB*, *psaC*, *psaI*, *psaJ*, *psaM*, *psbA*, *psbB*, *psbC*, *psbD*, *psbE*, *psbF*, *psbH*, *psbI*, *psbJ*, *psbK*, *psbL*, *psbM*, *psbN*, *psbT*, *psbZ*, *rbcL*, *rpl14*, *rpl16*, *rpl20*, *rpl21*, *rpl22*, *rpl23*, *rpl2*, *rpl32*, *rpl33*, *rpl36*, *rpoB*, *rpoC1*, *rpoC2*, *rps11*, *rps12*, *rps14*, *rps15*, *rps18*, *rps19*, *rps2*, *rps3*, *rps4*, *rps7*, *rps8*, *ycf12*, *ycf1*, *ycf2*, *ycf3*, *ycf4*, *ycf66*) was extracted from a total of 28 moss species, batch aligned with MAFFT (Katoh et al. 2005) as implemented in Geneious v10.0.2. The individual gene alignment was optimized with software Gblocks (Talavera and Castresana 2007) and concatenated with software FASconCAT-G (Kück and Longo 2014). The concatenated protein-coding gene dataset (Figshare database under DOI: https://doi.org/10.6084/m9.figshare.13363580.v1) was analyzed using software IQTREE2 (Quang et al. 2020) for phylogenetic reconstructions with the maximum-likelihood (ML) method and 1000 fast bootstrap replicates under the best-fit nucleotide substitution model (GTR + F+I + G4) as estimated by ModelFinder (Kalyaanamoorthy et al. 2017) and implemented in IQTREE2. The resulting phylogenetic tree (Figure 1) is consistent with previous phylogenomic studies of mosses. *C. aciphyllum* clustered with *Fissidens nobilis* from the same order Dicranales. The plastid sequence of the Antarctica moss *C. aciphyllum* would contribute to future research on the evolution and diversification of land plants.

**Figure 1.**
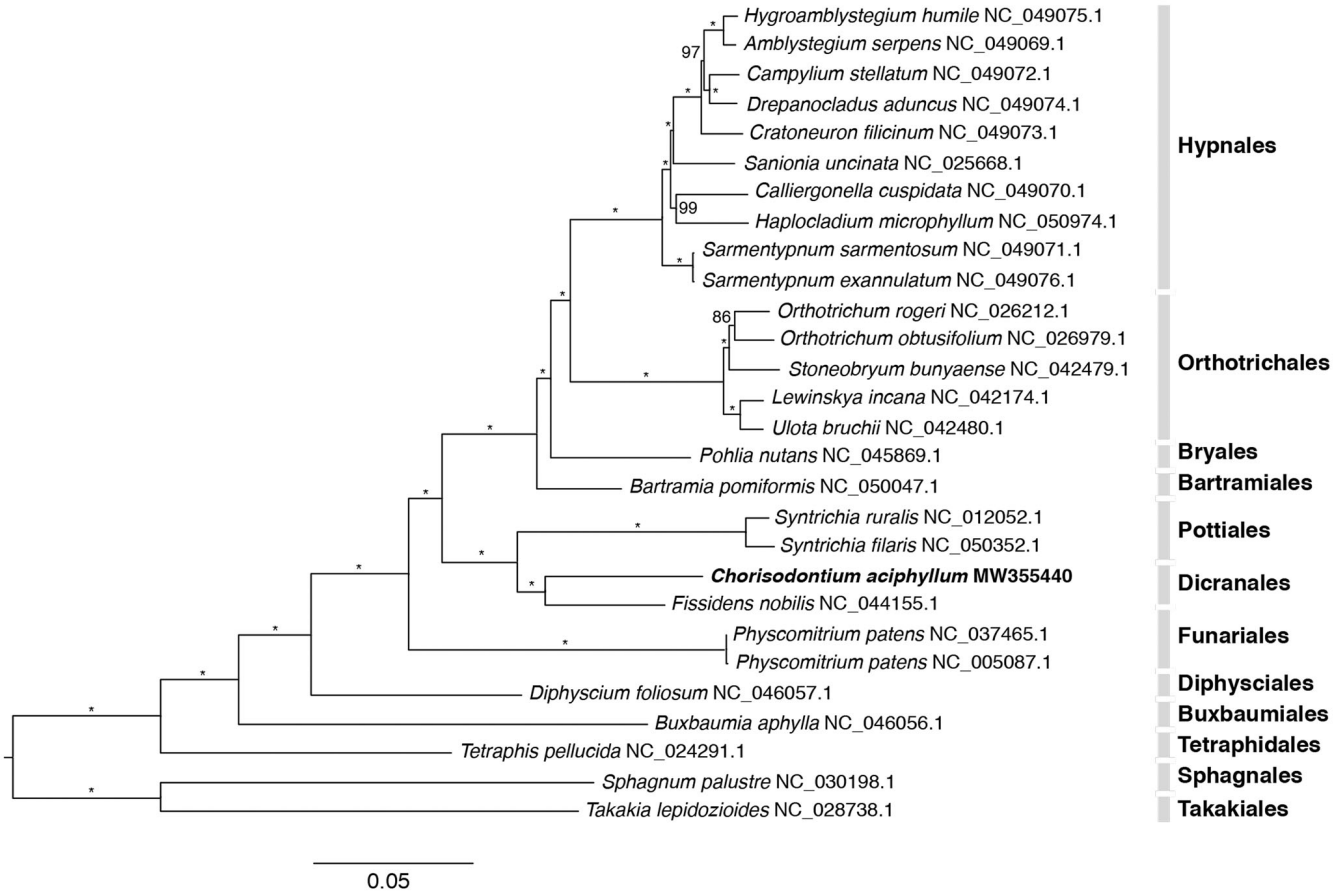
Phylogenetic position of *Chorisodontium aciphyllum* inferred using the maximum-likelihood method as implemented in IQTREE2 based on 81 protein-coding concatenated gene sequences. Ultra-fast bootstrap support values are based on 1000 replicates and are indicated above the branches. *100% bootstrap support.

## Data Availability

Chloroplast data supporting this study are openly available in GenBank at nucleotide database, https://www.ncbi.nlm.nih.gov/nuccore/MW355440, Associated BioProject, https://www.ncbi.nlm.nih.gov/bioproject/PRJNA684348, BioSample accession number at https://www.ncbi.nlm.nih.gov/biosample/SAMN16356356 and Sequence Read Archive at https://www.ncbi.nlm.nih.gov/sra/SRR13239595. The data matrix used for current phylogenetic reconstruction can be accessed from the Figshare database under DOI: https://doi.org/10.6084/m9.figshare.13363580.v1.
